# 呼吸道微生物在慢性阻塞性肺疾病与肺癌共病中的调控机制研究进展

**DOI:** 10.3779/j.issn.1009-3419.2025.106.33

**Published:** 2025-12-20

**Authors:** Yin ZHANG, Tianming ZHANG, Hong WANG

**Affiliations:** ^1^730030 兰州，兰州大学第二临床医学院; ^1^The Second Clinical Medical School of Lanzhou University, Lanzhou 730030, China; ^2^730030 兰州大学第二医院呼吸与危重症医学科; ^2^Department of Respiratory and Critical Care Medical, The Second Hospital of Lanzhou University, Lanzhou 730030, China

**Keywords:** 慢性阻塞性肺疾病, 肺肿瘤, 呼吸道微生物, 调控机制, Chronic obstructive pulmonary disease, Lung neoplasms, Respiratory microbiota, Regulatory mechanism

## Abstract

慢性阻塞性肺疾病（chronic obstructive pulmonary disease, COPD）与肺癌（lung carcinoma, LC）均为我国面临的重大公共卫生挑战。随着微生物检测方法的不断更新迭代，呼吸道微生物在呼吸系统疾病发生发展中的作用日益凸显。本文系统综述了COPD与LC病程中呼吸道微生物的动态特征，并探讨了其通过持续驱动慢性炎症、介导基因组损伤及参与免疫调控等机制在COPD-LC共病中发挥的关键桥梁作用，旨在深化对呼吸道微生物的认识，为优化现有治疗策略、探寻癌症早筛微生物标志物以及慢性呼吸系统疾病临床管理提供新视角。

## Abstract (en)

Both chronic obstructive pulmonary disease (COPD) and lung carcinoma (LC) are major public health challenges in China. With the continuous advancement of microbial detection methods, the role of the respiratory microbiome in the pathogenesis and progression of respiratory diseases has become increasingly prominent. This article systematically reviews the dynamic characteristics of the respiratory microbiome during the progression of COPD and LC, then explores its crucial bridging role in COPD-LC comorbidity through mechanisms such as persistently driving chronic inflammation, mediating genomic damage, and participating in immune regulation. The aim is to deepen the understanding of the respiratory microbiome, and to provide new perspectives for optimizing existing treatment strategies, exploring microbial biomarkers for early cancer screening, and improving the clinical management of chronic respiratory diseases.

慢性阻塞性肺疾病（chronic obstructive pulmonary disease, COPD）与肺癌（lung carcinoma, LC）均为全球范围内高发病率与死亡率的呼吸系统疾病。COPD现已成为全球第3位死亡原因，在中国约有9900万人患有该病，随着社会人口老龄化，COPD的患病率及疾病负担将继续上升^[1,2]^。LC是全球最常见的癌症之一，也是癌症相关死亡的主要原因^[3]^。近年来，诸多研究均证实COPD是LC发生发展的独立危险因素，COPD患者罹患LC的风险为正常人的3-6倍^[4,5]^，每年有0.8%-1.7%的COPD患者会发展为LC，约占COPD患者死亡原因的33%^[6]^，且调查显示共病患者5年生存率显著低于单纯LC患者^[7]^。

既往研究^[8]^中对COPD-LC共病机制的理解主要基于两者在危险因素、致病机制、遗传易感性、分子基因水平等方面存在的显著重叠。这些关联表明两者可能在一定程度上互为因果，相互诱发。近年来，随着微生物检测技术的不断迭代升级，呼吸道微生物作为一个动态的功能生态系统，其研究价值日益凸显。COPD与LC的独立研究均揭示了疾病过程中呼吸道微生物的显著失调，COPD患者呼吸道微生物的特征主要为菌群多样性降低、促炎菌属的富集等；而LC患者则在此基础上，进一步表现出与肿瘤组织病理类型和分期有关的微环境特异性菌群定植。COPD特征性的菌群失调已被证实不仅与疾病严重程度关联，还可能通过维持慢性炎症、诱导细胞基因突变、调控免疫等机制，直接参与LC的发生发展。本文通过分析微生物在两种疾病中的动态变化，探讨微生物如何通过持续驱动慢性炎症、直接诱导基因组损伤、免疫调控等机制，构成连接COPD与LC共病的重要生物学桥梁。

## 1 呼吸道微生物的构成

上呼吸道（upper respiratory tract, URT）的细菌负荷为下呼吸道（lower respiratory tract, LRT）的100-10,000倍，其中鼻部的优势属主要包括丙酸杆菌属、棒状杆菌属、葡萄球菌属和莫拉菌属，口腔优势属主要包括：普雷沃菌属、韦荣球菌属、链球菌属、嗜血杆菌属、梭杆菌属、奈瑟菌属和棒状杆菌属^[9]^。除细菌外，研究^[9]^表明健康人群URT还存在鼻病毒、博卡病毒、多瘤病毒、腺病毒、冠状病毒、曲霉属、青霉属、念珠菌属等病毒及真菌。健康的肺组织也拥有丰富的微生物组，在健康状态下，特定部位的微生物的构成主要由微生物的迁移与清除过程共同调控；而在疾病状态下，局部微环境及细菌繁殖率则成为影响微生物多样性的关键因素^[10]^。肺部微生物主要由细菌组成，其中优势门包括拟杆菌门、厚壁菌门、变形菌门、放线菌门；优势属包括假单胞菌属、链球菌属、普雷沃菌属、梭杆菌属和韦荣球菌属^[10]^。健康肺部还存在念珠菌属、马拉色菌属等真菌和少量病毒^[11]^。

## 2 COPD患者呼吸道微生物特征

COPD作为一种慢性炎症疾病，其特征是炎症反应不断破坏肺组织，导致气道狭窄、肺气肿以及持续的气流受限。该疾病的解剖学、生理学、免疫学特征及疾病治疗过程中使用的药物均可能对LRT的细菌群落施加选择压力，导致微生物群失调^[12]^。

Einarsson等^[13]^研究发现，COPD稳定期患者支气管肺泡灌洗液（bronchoalveolar lavage fluid, BALF）中微生物群的多样性显著低于健康吸烟者和非吸烟者。其中，假单胞菌相对丰度较高；而一些常见的厌氧菌，如普雷沃菌属、韦荣球菌属、放线菌属所占比例相对较低。Madapoosi等^[14]^发现，链球菌属、韦荣球菌属和奈瑟菌属的富集往往与第1秒用力呼气量（forced expiratory volume in one second, FEV₁）下降、COPD评估测试（COPD assessment test, CAT）评分升高及急性加重风险增加有关；而普雷沃菌属则显示出相反或有益的保护性作用。目前关于肺功能加速下降的机制仍知之甚少，Liang等^[15]^利用多组学研究发现，呼吸道长期携带金黄色葡萄球菌会导致气道同型半胱氨酸水平升高，进而激活宿主蛋白激酶B（protein kinase B, AKT1）信号通路，促使中性粒细胞的死亡方式从凋亡转变为中心粒细胞胞外诱捕网死亡，中性粒细胞的程序性凋亡对于及时清除病原体和炎症消退至关重要，而AKT1诱导的中性粒细胞增殖延长和凋亡延迟，会导致组织损伤、促炎因子释放，最终导致组织稳态环境受损。这种持续且未见缓解的慢性中性粒细胞炎症可能是COPD患者肺功能逐渐下降的驱动因素。

COPD急性加重（acute exacerbations of COPD, AECOPD）是导致患者肺功能下降和死亡的重要因素，利用16S核糖体RNA（16S ribosomal RNA gene, 16S rRNA）测序分析AECOPD患者的痰液样本^[16-18]^发现，急性加重期患者的呼吸道中含有数十至数百种不同的细菌群，与健康人群及疾病稳定期相比，嗜血杆菌属、莫拉菌属、克雷伯菌属、螺杆菌属丰度增高，这种变化与降钙素原（procalcitonin, PCT）和肿瘤坏死因子（tumor necrosis factor, TNF）水平升高、中性粒细胞外诱捕网复合物形成以及中性粒细胞和巨噬细胞介导的炎症反应程度均呈现显著关联。由此可见，COPD的加重与进展不能归因于单一病原体，优势菌属在疾病进展中的转化提示AECOPD可能与呼吸道微生物生态系统失衡有关。

尽管大多数研究集中在细菌组，但真菌组及病毒组也是微生物组的重要组成部分。对COPD患者的痰液样本^[19]^分析发现，气道真菌如酵母属与CAT评分升高相关，同时发现青霉属、曲霉属的富集是导致COPD患者频繁急性加重及死亡的独立风险因素。Bahetjan等^[20]^对70例AECOPD患者的BALF进行宏基因组下一代测序（metagenomic next-generation sequencing, mNGS）分析后表明，曲霉属与淋巴滤泡病毒属分别是AECOPD中最常见的真菌属与病毒属。有研究^[21]^发现呼吸道真菌的构成不仅与病情急性加重有关，还与特定的气道炎症表型有关，具体表现为在嗜酸性粒细胞性COPD中呼吸道曲霉属丰度升高，而非嗜酸性粒细胞性COPD中则多见红酵母属。van Rijn等^[22]^对COPD患者的呼吸道病毒进行分析发现，其主要构成包括鼻病毒、流感病毒、冠状病毒和副流感病毒。进一步研究^[23,24]^发现，鼻病毒、肠道病毒、流感病毒、肺炎衣原体与COPD的急性加重有关。

此外，微生物群多样性还会受到常用药物如吸入皮质类固醇（inhaled corticosteroids, ICS）的影响，研究^[25]^表明ICS可降低微生物群的α多样性，并可通过多途径促进气道细菌定植，包括诱导巨噬细胞表型转化从而降低其细菌清除能力、抑制抗菌肽（antimicrobial peptides, AMP）的产生以及抑制抗病毒干扰素反应，从而增加对继发细菌感染的易感性^[26]^，这些机制可共同导致呼吸道微生物失衡。但也有研究^[27]^认为ICS可以通过减轻炎症从而降低癌症相关风险。然而，其在LC预防中有效性的证据存在争议，多项研究^[28,29]^显示ICS可剂量依赖性地降低LC风险，而相关系统评价和荟萃分析未证实其显著益处。

抗生素作为AECOPD期间的主要治疗药物，适当的抗生素治疗可以缩短病程并减缓肺功能的进一步恶化，但区分细菌性与非细菌性感染依赖于高度敏感和特异的生物标志物，在实际临床工作中，可能存在部分由非细菌感染引起的AECOPD患者接受广谱抗生素治疗的情况。研究^[30]^表明，在AECOPD期间使用抗生素可能扰乱气道微生物群，降低微生物群的α多样性，进而影响肺部的免疫防御功能，这可能导致耐药性与炎症慢性化。因此，亟需一种更具靶向性的抗菌策略。近年来噬菌体疗法逐渐进入大众视野，其特异性裂解机制可在杀灭目标病原体的同时保全共生菌群，从而在控制感染的同时维护微生态稳定。此外，噬菌体还具备破坏生物膜、恢复细菌对抗生素敏感性的独特能力，这也是其在治疗细菌感染中展现潜力的关键优势^[31]^，为AECOPD的精准治疗提供了潜在新途径。

综上，ICS与抗生素的使用获益与风险并存，其应用需要根据不同COPD患者的具体情况，权衡利弊并进行个体化选择。

## 3 LC患者呼吸道微生物特征

恶性肿瘤的形成发展是一个涉及全身多处环境改变的长期过程，多项研究^[32,33]^一致观察到，与健康对照组或其癌旁组织相比，LC患者呼吸道微生物群的多样性发生显著改变，这提示微生物群失衡是LC的明确特征。

不同研究样本所揭示的微生物特征存在差异。在痰液或BALF样本中研究^[34]^更多报告了韦荣球菌属和巨球形菌属的富集；然而，一项研究^[35]^发现与健康对照相比，LC组织样本奈瑟菌属和链球菌属丰度升高，而葡萄球菌属丰度降低，且微生物群的α多样性呈现出健康组织→癌旁组织→癌组织的稳步下降趋势。这种差异可能表示肿瘤组织局部独特的微环境对菌群存在选择作用，也提示了根据样本类型解读微生物标志物的重要性。综合研究结果可以发现，链球菌属在LC发展中可能存在促进作用，而葡萄菌属则具有保护作用。

此外，不同组织病理学类型及分期的LC中存在不同的优势菌群。Yan等^[36]^对鳞状细胞癌（squamous cell carcinoma, SCC）、腺癌（adenocarcinoma, AC）的唾液进行16S rRNA测序结合定量聚合酶链式反应（quantitative polymerase chain reaction, qPCR）分析发现，SCC与AC患者中二氧化碳嗜纤维菌属、新月形单胞菌属、韦荣球菌属丰度显著升高，而奈瑟菌属丰度显著降低。链球菌属和卟啉单胞菌属的丰度仅在SCC患者中显著降低，而在AC中变化不显著。Leng等^[37]^对LC患者痰液进行微滴式数字PCR（droplet digital PCR, ddPCR）分析，发现痰液中食酸菌属、链球菌属、幽门螺杆菌及韦荣球菌属的丰度升高与SCC有关，二氧化碳嗜纤维菌属丰度的升高与AC有关。进一步研究^[38]^发现携带*TP53*突变的SCC患者中食酸菌属丰度升高。木杆菌属、真杆菌属和梭菌属被证实与小细胞肺癌（small cell lung carcinoma, SCLC）的发生存在正相关，而普雷沃菌属、反刍假丁酸弧菌可能与SCLC的发生呈负相关^[39]^。另有研究^[40]^表明栖热菌属在晚期（IIIB、IV期）患者的组织中丰度更高，而在发生转移的患者中军团菌属丰度更高。

基于16S rRNA测序研究证实了LC肿瘤细胞内存在微生物，Nejman等^[41]^发现LC具有自身独特的细菌群落，并通过组织学成像清晰地观察到细菌存在于癌细胞和免疫细胞内。进一步研究^[42]^表明，与T细胞、巨噬细胞等其他免疫细胞相比，肿瘤细胞中的细菌负荷显著升高。LC患者肿瘤细胞内的细菌可能通过β-catenin通路促进肿瘤生长，并形成免疫抵抗性的肿瘤微环境^[42]^。另有研究^[43]^表明肿瘤细胞内的细菌表位可被肿瘤细胞呈递并引发局部免疫反应，这一现象提示微生物源性抗原可能成为肿瘤新抗原的重要来源，并可通过改变肿瘤免疫微环境促进免疫编辑，但这一观点仍需更多的研究来证实。Liu等^[44]^对AC患者肿瘤组织的真菌组进行研究发现，肿瘤内存在聚多曲霉富集，并能够通过β-葡聚糖激活Dectin-1/CARD9通路，进而诱导髓源性抑制细胞的扩增和活化，从而塑造免疫抑制性肿瘤微环境，促进LC进展。这一研究首次明确肿瘤内存在活真菌，为将真菌纳入肿瘤预后生物标志物提供了有力证明。这些微生物的存在不仅构成了独特的肿瘤微环境，与肿瘤的发生发展密切相关，更已成为干预治疗反应、改善患者预后的潜在关键靶点。在胰腺癌中研究人员发现清除肿瘤内菌群可伴随程序性死亡受体1（programmed cell death 1, PD-1）表达上调。在对LC、结肠癌和胰腺癌患者的研究^[45]^中也证实，清除肿瘤微生物群能够增强肿瘤炎症反应、抑制肿瘤生长或改变对免疫原性肿瘤微环境的耐受性，从而改善抗肿瘤疗效。

针对非小细胞肺癌（non-small cell lung cancer, NSCLC）患者的荟萃分析^[46]^发现，在接受免疫检查点抑制剂（immune checkpoint inhibitors, ICIs）治疗前或治疗期间使用抗生素，会使患者的中位总生存期（overall survival, OS）缩短超过6个月；且进一步研究表明，在接受抗生素治疗的LC患者中，免疫相关不良事件（immune-related adverse events, irAEs）风险显著增加。这可能是由于抗生素使用引起的微生物多样性减少可调控与irAE有关的关键因素，进而增加irAE的发生风险^[47]^。与此同时，研究者^[48]^也发现乳杆菌属、双歧杆菌属等一些益生菌可能为宿主带来健康获益。有临床试验^[49]^发现口服含益生菌的酸奶对LC患者具有潜在保护作用。有研究^[50]^指出，益生菌可展现出与ICIs相当的抗肿瘤能力，两者联用几乎可以完全抑制小鼠体内肿瘤的生长。Jang等^[51]^通过对84例NSCLC患者的BALF进行16S rRNA基因测序，发现韦荣球菌属与程序性死亡配体1（programmed cell death ligand 1, PD-L1）高表达呈正相关，可能提示患者对免疫治疗反应较好；而奈瑟菌属则与PD-L1低表达有关。这表明特定呼吸道微生物或可作为预测免疫治疗预后的潜在生物学标志物，增加特定微生物丰度或可改善患者对免疫治疗的反应。

为阐明不同菌属在COPD、LC及其共病患者中的丰度变化规律，特将相关研究汇总见表1^[7,18,52-55]^。然而，现有研究中关于部分菌属的丰度变化趋势存在不一致甚至矛盾的结论，不一致性可能源于：（1）研究人群的异质性；（2）研究样本量差异；（3）疾病本身的动态演变；（4）治疗干预措施差异。期待未来开展的大样本、多中心、前瞻性研究为阐明COPD-LC共病中呼吸道微生物群的演变规律提供重要证据。

**表 1:** COPD、LC及共病患者呼吸道核心菌属丰度变化比较

Genus	COPD (FEV_1_/FVC<50%)	LC	COPD-LC	Specimen	Detection method
Pseudomonas^[52,53]^	↑	↑	↑/↓	BALF	mNGS/16S rRNA sequencing
Haemophilus^[7,52,53]^	↑	↑	↑	BALF	mNGS/16S rRNA sequencing
Veillonella^[7,52,53]^	↑	↑	↓	BALF	mNGS/16S rRNA sequencing
Actinomyces^[7,18]^	↑	↑	↓	BALF	mNGS/16S rRNA sequencing
Streptococcus^[7,52,53]^	↑	↑	↓	BALF	mNGS/16S rRNA sequencing
Rothia^[7,52,53]^	↑	↑	↓	BALF	mNGS/16S rRNA sequencing
Staphylococcus^[52,53]^	↑	↑/↓	NR	BALF	mNGS/16S rRNA sequencing
Neisseria^[7,52,53]^	↓	↑	↑	BALF	mNGS/16S rRNA sequencing
Prevotella^[7,54,55]^	↑/↓	↑	→	BALF	mNGS/16S rRNA sequencing

COPD: chronic obstructive pulmonary disease; LC: lung carcinoma; FEV_1_/FVC: forced expiratory volume in one second/forced vital capacity; BALF: bronchoalveolar lavage fluid; mNGS: metagenomic next-generation sequencing; 16S rRNA sequencing: 16S ribosomal RNA gene sequencing; ↑: increase; ↓: decrease; ↑/↓: inconsistent findings across studies; →: no significant change; NR: not reported.

## 4 微生物引起COPD-LC共病发病的调控机制

### 4.1 慢性炎症与免疫微环境重塑

慢性炎症是COPD与LC共有的病理特征之一。COPD患者气道和全身炎症程度均高于健康人群。呼吸道病毒感染会导致AMP降解，并刺激免疫细胞分泌干扰素-γ（interferon-γ, IFN-γ），从而抑制巨噬细胞和中性粒细胞对微生物的免疫反应，使微生物能够逃避气道免疫防御 。此外，吸入刺激物如烟草烟雾同样会导致AMP降解、巨噬细胞和中性粒细胞免疫功能受损，最终导致肺部免疫防御功能障碍。上述病理过程均会导致呼吸道持续性炎症状态，而炎症已被证实可以促进LC的发展^[56,57]^。研究^[58]^发现COPD样气道炎症会促进Kirsten大鼠肉瘤病毒癌基因同源物（Kirsten rat sarcoma viral oncogene homolog, *KRAS*）突变诱导的肺部肿瘤发生，这一过程与白细胞介素-6（interleukin-6, IL-6）水平的明显增加、信号转导与转录激活因子3（signal transducer and activator of transcription 3, STAT3）信号通路激活增加和髓系细胞反应扩增有关。Jungnickel等^[56]^研究表明，流感嗜血杆菌在引发肺部炎症的同时会激活Toll样受体2（Toll-like receptor 2, TLR2）和TLR4信号通路，在携带致癌基因*KRAS*的转基因小鼠模型中，这种TLR通路的激活会促进KRAS诱导的早期AC生长。证据^[59]^表明LRT中普雷沃菌属、韦荣球菌属和链球菌属丰度增加，会伴随炎症细胞浸润加剧以及细胞外信号调节激酶（extracellular signal‐regulated kinase, ERK）和磷脂酰肌醇3-激酶（phosphoinositide 3-kinase, PI3K）信号通路表达上调，其中PI3K可参与调节细胞增殖和存活，是参与NSCLC发病机制的关键通路。体外实验^[60]^进一步表明，当香烟烟雾与不可分型流感嗜血杆菌共同作用于气道上皮细胞，可进一步上调PI3K、ERK及血管内皮生长因子（vascular endothelial growth factor, VEGF）信号通路，这表明两者在驱动癌症发生过程中具有协同作用。Tsay等^[60]^认为LRT菌群失衡与I-IIIA期患者生存率降低以及IIIB-IV期患者经实体肿瘤疗效评价标准（Response Evaluation Criteria in Solid Tumors, RECIST）评估的肿瘤进展恶化有关。他们同样发现这种菌群失衡可通过引起IL-17、PI3K、丝裂原活化蛋白激酶（mitogen-activated protein kinase, MAPK）和ERK通路表达上调，从而导致慢性炎症、Th17细胞分化增强以及PD-L1表达增加，最终导致肿瘤的发生发展。

局部微生物失衡会激活肺驻留的γδT（gammadelta T）细胞，Jin等^[61]^研究发现在LC模型中“无菌小鼠”（germ-free mice, GF）肺部的γδT细胞丰度显著低于“无特定病原体小鼠”（specific pathogen free mice, SPF）。这表明正常呼吸道微生物可维持γδT细胞丰度，从而保护呼吸道黏膜。然而，当局部微生物失调时，则可刺激骨髓细胞产生IL-1β和IL-23，进而诱导肺部免疫细胞Vγ6^+^Vδ1^+^γδT的增殖和活化，活化的γδT细胞会产生大量IL-17和其他促炎介质，以促进中性粒细胞浸润和肿瘤细胞增殖。

综上，菌群失调可能扰乱肺部免疫稳态：一方面，微生物多样性下降会影响抗原呈递细胞的初始活化过程，从而削弱其对肿瘤抗原的应答能力^[62]^；另一方面，特定细菌过度增殖又可能过度激活免疫系统，引发CD4^+^ T细胞异常增殖、部分信号通路表达上调等，这些机制共同参与了肿瘤的发展^[63]^。

### 4.2 基因组损伤

研究^[64]^表明气道微生物群与支气管上皮基因表达之间存在联系。微生物可持续驱动COPD气道慢性炎症，并通过基因毒素或特异性代谢物直接导致呼吸道上皮细胞DNA受损或突变，从而启动或促进致癌过程^[65]^。铜绿假单胞菌感染后可通过其III型分泌系统向宿主细胞递送ExoS毒素，可激活宿主ATM激酶，导致组蛋白H2AX磷酸化，进而诱导DNA双链断裂和修复灶形成^[66]^。一些细菌毒素，包括细胞毒性坏死因子1（cytotoxic necrotizing factor 1, CNF1）、细胞溶解毒素、脆弱拟杆菌毒素以及细菌驱动的硫化氢和超氧自由基，也已被确定会导致DNA损伤进而导致肿瘤发生^[32]^。诸如此类，造成基因不稳定的微生物均有可能成为潜在的药物设计靶点，以干预感染有关的DNA损伤。

菌群失调还被认为会增加肺部与DNA损伤有关的活性氧（reactive oxygen species, ROS）含量，ROS会直接损伤DNA导致致癌突变。同时ROS还可以通过激活核因子κB（nuclear factor-κB, NF-κB）通路，诱导多种促炎因子的释放，如肿瘤坏死因子-α（tumor necrosis factor-α, TNF-α）、IL-6和IL-8。NF-κB的激活进一步加剧了局部炎症反应。氧化应激与慢性炎症在COPD和LC的进展中可相互促进形成恶性循环，导致气道和肺组织持续损伤，为LC发生发展创造了关键微环境^[67]^。

表观遗传机制也可以将COPD与LC发生联系起来，与直接基因突变不同的是，基因的表观遗传变化通常会导致细胞内RNA和蛋白质表达水平变化，从而导致癌症发生^[68]^。细菌感染通过调控宿主表观基因组以存活、复制并入侵宿主先天免疫反应，例如肺炎球菌可产生肺炎溶素和丙酮酸氧化酶等毒力因子，利用宿主PP1磷酸酶使组蛋白H3第10位丝氨酸去磷酸化，从而促进细菌定植与增殖^[69]^。鉴于H3S10ph水平升高与癌症的发生与扩散有关^[70]^，这一发现为探索共生链球菌在癌症发生中的保护性角色提供了全新视角。

肿瘤中还发现了一种严格产生*L*-乳酸的乳酸菌——惰性乳杆菌，在NSCLC中通过产生*L*-乳酸，启动肿瘤细胞的代谢重编程，增强其对放化疗的抵抗能力，研究^[71]^表明其与NSCLC的无进展生存期缩短密切关联。然而，该领域的实验证据仍相对匮乏，亟需未来研究进一步探索。

### 4.3 免疫调控

呼吸道微生物被认为是人类免疫系统不可或缺的一部分。当人体物理屏障功能缺损或免疫监视失效时，呼吸道微生物组的组成结构将发生紊乱，细菌易位增加，由此引发链式反应，最终或导致癌症发生^[72]^。

如上文所述，变形菌门的丰度与COPD患者疾病的严重程度呈正相关，变形菌门中两种普遍存在的机会致病性病原体即摩氏摩根菌和弗格森埃希菌可通过产生不同的毒力因子发挥免疫刺激作用^[73]^。已有研究^[74]^认为细菌驱动的免疫激活可能会加剧致瘤性炎症。为了防止因吸入颗粒物而引发过度的炎症反应，肺部在正常情况下会维持较高的免疫耐受性。在此基础上实验发现雾化吸入抗生素在引起呼吸道细菌载量降低的同时，还会引起呼吸道调节性T细胞（regulatory T cells, Treg）减少及自然杀伤（natural killer, NK）细胞的活化增强，上述机制可减轻微环境中的免疫抑制状态，使得肿瘤肺部转移显著减少^[73]^。免疫细胞的功能异常会加剧氧化应激，进而增加LC进展风险^[67]^。有研究^[75]^发现，在致癌物诱导的小鼠AC模型中，雾化吸入鼠李糖乳杆菌可减少LC的发展。另有研究发现一些特殊微生物可通过调节局部代谢和免疫促进肿瘤发生，如Hosgood等^[76]^发现定植于口腔或肺部的细菌具有代谢和解毒多环芳烃（polycyclic aromatic hydrocarbons, PAHs）的能力，表明呼吸道微生物群可能通过调节宿主解毒功能影响PAHs相关LC的发生发展。

这些发现表明细菌在调控抗肿瘤免疫反应中具有重要作用，并表明呼吸道细菌稳态对机体抗肿瘤免疫反应至关重要。呼吸道微生物群通过影响局部免疫与代谢稳态参与LC发生，未来研究需深入解析关键菌种的作用机制，并探索以菌群为靶点的精准防治新策略。

综上，呼吸道微生物群失衡与COPD、LC及共病的发生发展之间的关系是双向的。呼吸道微生物紊乱进而引起的一系列生理反应是COPD-LC共病机制中的核心病理桥梁（图1），且各个机制之间互为因果，相互影响。

**图 1:**
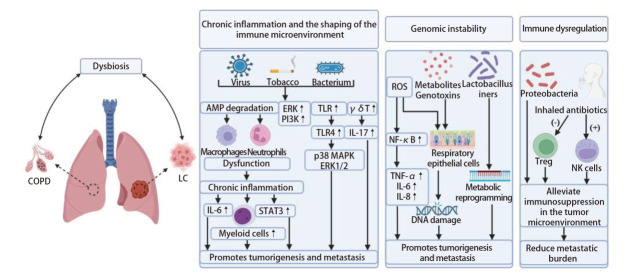
微生物引起COPD-LC共病发病的调控机制

## 5 总结及展望

值得深入探讨的是，上述“桥梁”并非处于静态，而是一个动态演变的微生态-宿主互作网络，可能随着疾病分期、治疗干预而发生转变，从而持续驱动共病的恶性进展。如上文所述，微生物在癌症治疗中可发挥激动剂或抑制剂的双重作用，这取决于治疗策略，在之后的研究中需要对相关机制进行更深入的讨论。肺部微生物作为新型研究领域，相关研究有限，现有的关于LC特异性微生物组组成的报告并不一致，而可能导致不一致的原因包括样本类型、采样方法、检测方式以及不同地区、不同人种差异。因此，识别和确定健康及患病个体肺部微生物组的组成和丰度仍然是一个重大挑战。

展望未来，亟需高灵敏度、高分辨率微生物检测技术，以系统解析肺部微生物的空间异质性及其与宿主细胞间的互作模式，以便进一步筛选具有代表性的微生物标志物，实现疾病早筛及精准干预。还可以将微生物组学与代谢组、蛋白组等多维数据整合，构建COPD-LC的多组学联合风险预测及诊断模型。通过形成“检测-诊断-治疗-评估”的精准医学体系，呼吸道微生物组研究有望为COPD-LC早期预警和精准防治提供新的理论依据和技术路径。

## References

[1] Adeloye D, Song P, Zhu Y, et al. Global, regional, and national prevalence of, and risk factors for, chronic obstructive pulmonary disease (COPD) in 2019: a systematic review and modelling analysis. Lancet Respir Med, 2022, 10(5): 447-458. doi: 10.1016/S2213-2600(21)00511-7 35279265 PMC9050565

[2] Liang Y, Sun Y. COPD in China: current status and challenges. Arch Bronconeumol, 2022, 58(12): 790-791. doi: 10.1016/j.arbres.2022.04.001 35581046

[3] Thai AA, Solomon BJ, Sequist LV, et al. Lung cancer. Lancet, 2021, 398(10299): 535-554. doi: 10.1016/S0140-6736(21)00312-3 34273294

[4] Perrotta F, D’Agnano V, Scialò F, et al. Evolving concepts in COPD and lung cancer: a narrative review. Minerva Med, 2022, 113(3): 436-448. doi: 10.23736/S0026-4806.22.07962-9 35156786

[5] Chen H, Hu XB, Zhou J, et al. Association of chronic obstructive pulmonary disease with risk of lung cancer in individuals aged 40 years and older: a cross-sectional study based on NHANES 2013-2018. PLoS One, 2024, 19(10): e0311537. doi: 10.1371/journal.pone.0311537 39441785 PMC11498685

[6] Gu Z, Wu Y, Yu F, et al. Integrating genetic and clinical data to predict lung cancer in patients with chronic obstructive pulmonary disease. BMC Pulm Med, 2024, 24(1): 618. doi: 10.1186/s12890-024-03444-5 39696223 PMC11656926

[7] He JQ, Chen Q, Wu SJ, et al. Potential implications of the lung microbiota in patients with chronic obstruction pulmonary disease and non-small cell lung cancer. Front Cell Infect Microbiol, 2022, 12: 937864. doi: 10.3389/fcimb.2022.937864 35967848 PMC9363884

[8] Zhou C, Qin Y, Zhao W, et al. International expert consensus on diagnosis and treatment of lung cancer complicated by chronic obstructive pulmonary disease. Transl Lung Cancer Res, 2023, 12(8): 1661-1701. doi: 10.21037/tlcr-23-339 37691866 PMC10483081

[9] Zhao L, Luo JL, Ali MK, et al. The human respiratory microbiome: current understandings and future directions. Am J Respir Cell Mol Biol, 2023, 68(3): 245-255. doi: 10.1165/rcmb.2022-0208TR 36476129 PMC9989478

[10] Zhu Y, Chang D. Interactions between the lung microbiome and host immunity in chronic obstructive pulmonary disease. Chronic Dis Transl Med, 2023, 9(2): 104-121. doi: 10.1002/cdt3.66 37305112 PMC10249200

[11] Martinsen EMH, Eagan TML, Leiten EO, et al. The pulmonary mycobiome-a study of subjects with and without chronic obstructive pulmonary disease. PLoS One, 2021, 16(4): e0248967. doi: 10.1371/journal.pone.0248967 33826639 PMC8026037

[12] Lipinksi JH, Ranjan P, Dickson RP, et al. The lung microbiome. J Immunol, 2024, 212(8): 1269-1275. doi: 10.4049/jimmunol.2300716 38560811 PMC11073614

[13] Einarsson GG, Comer DM, McIlreavey L, et al. Community dynamics and the lower airway microbiota in stable chronic obstructive pulmonary disease, smokers and healthy non-smokers. Thorax, 2016, 71(9): 795-803. doi: 10.1136/thoraxjnl-2015-207235 27146202

[14] Madapoosi SS, Cruickshank-Quinn C, Opron K, et al. Lung microbiota and metabolites collectively associate with clinical outcomes in milder stage chronic obstructive pulmonary disease. Am J Respir Crit Care Med, 2022, 206(4): 427-439. doi: 10.1164/rccm.202110-2241OC 35536732 PMC11418810

[15] Liang W, Yang Y, Gong S, et al. Airway dysbiosis accelerates lung function decline in chronic obstructive pulmonary disease. Cell Host Microbe, 2023, 31(6): 1054-1070.e9. doi: 10.1016/j.chom.2023.04.018 37207649

[16] Tangedal S, Nielsen R, Aanerud M, et al. Sputum microbiota and inflammation at stable state and during exacerbations in a cohort of chronic obstructive pulmonary disease (COPD) patients. PLoS One, 2019, 14(9): e0222449. doi: 10.1371/journal.pone.0222449 31527888 PMC6748569

[17] Xue Q, Xie Y, He Y, et al. Lung microbiome and cytokine profiles in different disease states of COPD: a cohort study. Sci Rep, 2023, 13(1): 5715. doi: 10.1038/s41598-023-32901-0 37029178 PMC10080507

[18] Stankovic MM. Lung microbiota: from healthy lungs to development of chronic obstructive pulmonary disease. Int J Mol Sci, 2025, 26(4): 1403. doi: 10.3390/ijms26041403 40003871 PMC11854937

[19] Tiew PY, Dicker AJ, Keir HR, et al. A high-risk airway mycobiome is associated with frequent exacerbation and mortality in COPD. Eur Respir J, 2021, 57(3): 2002050. doi: 10.1183/13993003.02050-2020 32972986

[20] Bahetjan K, Yu-Xia, Lin S, et al. Analysis of the bronchoalveolar lavage fluid microbial flora in COPD patients at different lung function during acute exacerbation. Sci Rep, 2025, 15(1): 13179. doi: 10.1038/s41598-025-96746-5 40240456 PMC12003667

[21] Tiew PY, Thng KX, Chotirmall SH. Clinical *Aspergillus* signatures in COPD and bronchiectasis. J Fungi, 2022, 8(5): 480. doi: 10.3390/jof8050480 PMC9146266 35628736

[22] van Rijn AL, van Boheemen S, Sidorov I, et al. The respiratory virome and exacerbations in patients with chronic obstructive pulmonary disease. PLoS One, 2019, 14(10): e0223952. doi: 10.1371/journal.pone.0223952 31647831 PMC6812800

[23] Bowerman KL, Rehman SF, Vaughan A, et al. Disease-associated gut microbiome and metabolome changes in patients with chronic obstructive pulmonary disease. Nat Commun, 2020, 11(1): 5886. doi: 10.1038/s41467-020-19701-0 33208745 PMC7676259

[24] Leung JM, Tiew PY, Mac Aogáin M, et al. The role of acute and chronic respiratory colonization and infections in the pathogenesis of COPD. Respirology, 2017, 22(4): 634-650. doi: 10.1111/resp.13032 28342288 PMC7169176

[25] Chen L, Yang M, Wang Q, et al. Airway microbial and metabolic features associated with ICS therapy in COPD. Front Pharmacol, 2025, 16: 1714879. doi: 10.3389/fphar.2025.1714879 41409595 PMC12705548

[26] Lea S, Higham A, Beech A, et al. How inhaled corticosteroids target inflammation in COPD. Eur Respir Rev, 2023, 32(170): 230084. doi: 10.1183/16000617.0084-2023 37852657 PMC10582931

[27] Ge F, Feng Y, Huo Z, et al. Inhaled corticosteroids and risk of lung cancer among chronic obstructive pulmonary disease patients: a comprehensive analysis of nine prospective cohorts. Transl Lung Cancer Res, 2021, 10(3): 1266-1276. doi: 10.21037/tlcr-20-1126 33889508 PMC8044471

[28] Forder A, Zhuang R, Souza VGP, et al. Mechanisms contributing to the comorbidity of COPD and lung cancer. Int J Mol Sci, 2023, 24(3): 2859. doi: 10.3390/ijms24032859 36769181 PMC9918127

[29] Raymakers AJN, Sadatsafavi M, Sin DD, et al. Inhaled corticosteroids and the risk of lung cancer in COPD: a population-based cohort study. Eur Respir J, 2019, 53(6): 1801257. doi: 10.1183/13993003.01257-2018 30956205

[30] Luo L, Tang J, Du X, et al. Chronic obstructive pulmonary disease and the airway microbiome: a review for clinicians. Respir Med, 2024, 225: 107586. doi: 10.1016/j.rmed.2024.107586 38460708

[31] Sarkodie-Addo P, Osman AH, Aglomasa BC, et al. Phage therapy in the management of respiratory and pulmonary infections: a systematic review. Ther Adv Infect Dis, 2025, 12: 20499361241307841. doi: 10.1177/20499361241307841 PMC11760135 39866829

[32] Li R, Li J, Zhou X. Lung microbiome: new insights into the pathogenesis of respiratory diseases. Signal Transduct Target Ther, 2024, 9(1): 19. doi: 10.1038/s41392-023-01722-y 38228603 PMC10791971

[33] Kanteres T, Angelakopoulou IM, Angelakopoulou EA, et al. Lung microbiome dynamics in health and lung cancer. Microb Genom, 2025, 11(10): 001509. doi: 10.1099/mgen.0.001509 41055637 PMC12503387

[34] Lee SH, Sung JY, Yong D, et al. Characterization of microbiome in bronchoalveolar lavage fluid of patients with lung cancer comparing with benign mass like lesions. Lung Cancer, 2016, 102: 89-95. doi: 10.1016/j.lungcan.2016.10.016 27987594

[35] Liu HX, Tao LL, Zhang J, et al. Difference of lower airway microbiome in bilateral protected specimen brush between lung cancer patients with unilateral lobar masses and control subjects. Int J Cancer, 2018, 142(4): 769-778. doi: 10.1002/ijc.31098 29023689

[36] Yan X, Yang M, Liu J, et al. Discovery and validation of potential bacterial biomarkers for lung cancer. Am J Cancer Res, 2015, 5(10): 3111-3122. 26693063 PMC4656734

[37] Leng Q, Holden VK, Deepak J, et al. Microbiota biomarkers for lung cancer. Diagnostics (Basel), 2021, 11(3): 407. doi: 10.3390/diagnostics11030407 33673596 PMC7997424

[38] Greathouse KL, White JR, Vargas AJ, et al. Interaction between the microbiome and TP 53 in human lung cancer. Genome Biol, 2018, 19(1): 123. doi: 10.1186/s13059-018-1501-6 30143034 PMC6109311

[39] Sun Y, Wen M, Liu Y, et al. The human microbiome: a promising target for lung cancer treatment. Front Immunol, 2023, 14: 1091165. doi: 10.3389/fimmu.2023.1091165 36817461 PMC9936316

[40] Yu G, Gail MH, Consonni D, et al. Characterizing human lung tissue microbiota and its relationship to epidemiological and clinical features. Genome Biol, 2016, 17(1): 163. doi: 10.1186/s13059-016-1021-1 27468850 PMC4964003

[41] Nejman D, Livyatan I, Fuks G, et al. The human tumor microbiome is composed of tumor type-specific intracellular bacteria. Science, 2020, 368(6494): 973-980. doi: 10.1126/science.aay9189 32467386 PMC7757858

[42] Wong-Rolle A, Dong Q, Zhu Y, et al. Spatial meta-transcriptomics reveal associations of intratumor bacteria burden with lung cancer cells showing a distinct oncogenic signature. J Immunother Cancer, 2022, 10(7): e004698. doi: 10.1136/jitc-2022-004698 35793869 PMC9260850

[43] Kalaora S, Nagler A, Nejman D, et al. Identification of bacteria-derived HLA-bound peptides in melanoma. Nature, 2021, 592(7852): 138-143. doi: 10.1038/s41586-021-03368-8 33731925 PMC9717498

[44] Liu NN, Yi CX, Wei LQ, et al. The intratumor mycobiome promotes lung cancer progression via myeloid-derived suppressor cells. Cancer Cell, 2023, 41(11): 1927-1944.e9. doi: 10.1016/j.ccell.2023.08.012 37738973

[45] Yang L, Li A, Wang Y, et al. Intratumoral microbiota: roles in cancer initiation, development and therapeutic efficacy. Signal Transduct Target Ther, 2023, 8(1): 35. doi: 10.1038/s41392-022-01304-4 36646684 PMC9842669

[46] Lurienne L, Cervesi J, Duhalde L, et al. NSCLC immunotherapy efficacy and antibiotic use: a systematic review and *meta*-analysis. J Thorac Oncol, 2020, 15(7): 1147-1159. doi: 10.1016/j.jtho.2020.03.002 32173463

[47] Jing Y, Chen X, Li K, et al. Association of antibiotic treatment with immune-related adverse events in patients with cancer receiving immunotherapy. J Immunother Cancer, 2022, 10(1): e003779. doi: 10.1136/jitc-2021-003779 35058327 PMC8772460

[48] Du T, Lei A, Zhang N, et al. The beneficial role of probiotic *Lactobacillus* in respiratory diseases. Front Immunol, 2022, 13: 908010. doi: 10.3389/fimmu.2022.908010 35711436 PMC9194447

[49] Yang JJ, Yu D, Xiang YB, et al. Association of dietary fiber and yogurt consumption with lung cancer risk: a pooled analysis. JAMA Oncol, 2020, 6(2): e194107. doi: 10.1001/jamaoncol.2019.4107 31647500 PMC6813596

[50] Sivan A, Corrales L, Hubert N, et al. Commensal bifidobacterium promotes antitumor immunity and facilitates anti-PD-L1 efficacy. Science, 2015, 350 (6264): 1084-1089. doi: 10.1126/science.aac4255 26541606 PMC4873287

[51] Jang HJ, Choi JY, Kim K, et al. Relationship of the lung microbiome with PD-L 1 expression and immunotherapy response in lung cancer. Respir Res, 2021, 22(1): 322. doi: 10.1186/s12931-021-01919-1 34963470 PMC8715618

[52] Wu J, Zhang Y, Duan J, et al. A metagenomic next-generation sequencing (mNGS)-based analysis of bronchoalveolar lavage samples in patients with an acute exacerbation of chronic obstructive pulmonary disease. J Mol Histol, 2024, 55(5): 709-719. doi: 10.1007/s10735-024-10225-1 39060894

[53] Wang J, Su W, Chen Q, et al. Microbiome-metabolome dysbiosis of bronchoalveolar lavage fluid of lung cancer patients. Front Microbiol, 2025, 16: 1669172. doi: 10.3389/fmicb.2025.1669172 41311499 PMC12647101

[54] Chen Q, Hou K, Tang M, et al. Screening of potential microbial markers for lung cancer using metagenomic sequencing. Cancer Med, 2023, 12(6): 7127-7139. doi: 10.1002/cam4.5513 36480163 PMC10067086

[55] Lin Z, Jiang Y, Liu H, et al. Airway microbiota and immunity associated with chronic obstructive pulmonary disease severity. J Transl Med, 2025, 23(1): 962. doi: 10.1186/s12967-025-06986-2 40859318 PMC12382185

[56] Jungnickel C, Schnabel PA, Bohle R, et al. Nontypeable haemophilus influenzae-promoted proliferation of Kras-induced early adenomatous lesions is completely dependent on Toll-like receptor signaling. Am J Pathol, 2017, 187(5): 973-979. doi: 10.1016/j.ajpath.2017.01.003 28279655

[57] Li XY, Zhou JJ, Zhao CT, et al. Research progress of tumor-associated neutrophilsin the occurrence and development of lung cancer. Zhongguo Feiai Zazhi, 2025, 28(1): 55-62. 39988440 10.3779/j.issn.1009-3419.2025.101.02 PMC11848648

[58] Caetano MS, Zhang H, Cumpian AM, et al. IL-6 blockade reprograms the lung tumor microenvironment to limit the development and progression of *K-ras* mutant lung cancer. Cancer Res, 2016, 76(11): 3189-3199. doi: 10.1158/0008-5472.CAN-15-2840 27197187 PMC4891282

[59] Tsay JJ, Wu BG, Badri MH, et al. Airway microbiota is associated with upregulation of the PI3K pathway in lung cancer. Am J Respir Crit Care Med, 2018, 198(9): 1188-1198. doi: 10.1164/rccm.201710-2118OC 29864375 PMC6221574

[60] Tsay JCJ, Wu BG, Sulaiman I, et al. Lower airway dysbiosis affects lung cancer progression. Cancer Discov, 2021, 11(2): 293-307. doi: 10.1158/2159-8290.CD-20-0263 33177060 PMC7858243

[61] Jin C, Lagoudas GK, Zhao C, et al. Commensal microbiota promote lung cancer development via γδ T cells. Cell, 2019, 176(5): 998-1013.e16. doi: 10.1016/j.cell.2018.12.040 30712876 PMC6691977

[62] Hou K, Wu ZX, Chen XY, et al. Microbiota in health and diseases. Signal Transduct Target Ther, 2022, 7(1): 135. doi: 10.1038/s41392-022-00974-4 35461318 PMC9034083

[63] Mills KHG. IL-17 and IL-17-producing cells in protection versus pathology. Nat Rev Immunol, 2023, 23(1): 38-54. doi: 10.1038/s41577-022-00746-9 35790881 PMC9255545

[64] Ramsheh MY, Haldar K, Esteve-Codina A, et al. Lung microbiome composition and bronchial epithelial gene expression in patients with COPD versus healthy individuals: a bacterial 16S rRNA gene sequencing and host transcriptomic analysis. Lancet Microbe, 2021, 2(7): e300-e310. doi: 10.1016/S2666-5247(21)00035-5 35544166

[65] Li B, Wang D, Zhang C, et al. Role of respiratory system microbiota in development of lung cancer and clinical application. Imeta, 2024, 3(5): e232. doi: 10.1002/imt2.232 39429871 PMC11488069

[66] Elsen S, Collin-Faure V, Gidrol X, et al. The opportunistic pathogen pseudomonas aeruginosa activates the DNA double-strand break signaling and repair pathway in infected cells. Cell Mol Life Sci, 2013, 70(22): 4385-4397. doi: 10.1007/s00018-013-1392-3 23760206 PMC11113669

[67] Jiang H, Huang G, Feng D, et al. Comorbidity of lung cancer and chronic obstructive pulmonary disease: correlation and optimization of treatment strategies. Transl Lung Cancer Res, 2025, 14(6): 2296-2308. doi: 10.21037/tlcr-2025-480 40673092 PMC12261252

[68] Recillas-Targa F. Cancer epigenetics: an overview. Arch Med Res, 2022, 53(8): 732-740. doi: 10.1016/j.arcmed.2022.11.003 36411173

[69] Dong W, Rasid O, Chevalier C, et al. Streptococcus pneumoniae infection promotes histone H3 dephosphorylation by modulating host PP1 phosphatase. Cell Rep, 2020, 30(12): 4016-4026.e4. doi: 10.1016/j.celrep.2020.02.116 32209465

[70] Komar D, Juszczynski P. Rebelled epigenome: histone H3S10 phosphorylation and H3S10 kinases in cancer biology and therapy. Clin Epigenetics, 2020, 12(1): 147. doi: 10.1186/s13148-020-00941-2 33054831 PMC7556946

[71] Colbert LE, El Alam MB, Wang R, et al. Tumor-resident *lactobacillus* iners confer chemoradiation resistance through lactate-induced metabolic rewiring. Cancer Cell, 2023, 41(11): 1945-1962.e11. doi: 10.1016/j.ccell.2023.09.012 37863066 PMC10841640

[72] Mao Q, Jiang F, Yin R, et al. Interplay between the lung microbiome and lung cancer. Cancer Lett, 2018, 415: 40-48. doi: 10.1016/j.canlet.2017.11.036 29197615

[73] Le Noci V, Guglielmetti S, Arioli S, et al. Modulation of pulmonary microbiota by antibiotic or probiotic aerosol therapy: a strategy to promote immunosurveillance against lung metastases. Cell Rep, 2018, 24(13): 3528-3538. doi: 10.1016/j.celrep.2018.08.090 30257213

[74] Ramírez-Labrada AG, Isla D, Artal A, et al. The influence of lung microbiota on lung carcinogenesis, immunity, and immunotherapy. Trends Cancer, 2020, 6(2): 86-97. doi: 10.1016/j.trecan.2019.12.007 32061309

[75] Le Noci V, Bernardo G, Manenti G, et al. Live or heat-killed *Lactobacillus rhamnosus* aerosolization decreases adenomatous lung cancer development in a mouse carcinogen-induced tumor model. Int J Mol Sci, 2022, 23(21): 12748. doi: 10.3390/ijms232112748 36361537 PMC9656640

[76] Hosgood HD 3^rd^ , Sapkota AR, Rothman N, et al. The potential role of lung microbiota in lung cancer attributed to household coal burning exposures. Environ Mol Mutagen, 2014, 55(8): 643-651. doi: 10.1002/em.21878 24895247 PMC4217127

